# Association Between Prior Antipsychotic Adherence and Adherence Three Years After Clozapine Initiation: A Real-World Observational Study

**DOI:** 10.1192/bjo.2024.101

**Published:** 2024-08-01

**Authors:** Sébastien Brodeur, Josiane Courteau, Alain Vanasse, Marc-André Roy, Mireille Courteau

**Affiliations:** 1 Laval University, Faculty of Medicine, Quebec City, Canada; 2 Institute of Psychiatry, Psychology & Neuroscience, King's College London, London, United Kingdom; 3 PRIMUS Group, Sherbrooke, Canada; 4 CHUS Research Center, Sherbrooke, Canada; 5 Sherbrooke University, Sherbrooke, Canada; 6 Laval University, Quebec City, Canada

## Abstract

**Aims:**

Our previous findings challenged the widely held view among a large proportion of psychiatrists (41% to 82%) that previous non-adherence to antipsychotics is a major barrier to the introduction of clozapine (Brodeur et al. 2022 BJPsych). Indeed, our previous work showed that most patients, even those with the poorest adherence profiles, remained on their treatment after clozapine initiation (>68% for clozapine and >84% for all antipsychotics combined) after one year of follow-up. Because of this, the study extended the follow-up period to three years to assess whether patterns of adherence were sustained over time. Therefore, this study aimed to determine whether poor adherence prior to initiating clozapine predicted poor adherence to clozapine or any other antipsychotic (including clozapine) three years after initiation.

**Methods:**

This cohort study included 2,258 patients living in Quebec (Canada) with a diagnosis of SCZ who initiated oral clozapine between 2009 and 2016 (index date). Adherence to AP was measured by the medication possession ratio (MPR) over a 1-year period before and a 3-year period after the index date. Five groups of patients were formed based on their prior MPR level (independent variable), and two dependent variables were defined after clozapine initiation (good adherence (MPR ≥ 0.8) to any APs and to clozapine only). In addition to multiple logistic regression, state sequence analysis was used to visualise the trajectories of AP use over time, before and after clozapine initiation, for each group.

**Results:**

The graphical representation of the SSA immediately showed that AP adherence was significantly improved in all groups, regardless of the level of previous adherence to AP treatment. On the other hand, logistic regression showed that poorer adherence to APs before the index date was significantly associated with an increased risk of poor adherence to any AP treatment 3 years after the index date (adjusted ORs ranging from 2.2 to 3.0). However, the majority of patients (ranging from 80.8% to 92.4%) had good adherence to any APs and to oral clozapine (ranging from 57.7% to 73.8%), regardless of previous adherence level.

**Conclusion:**

These results add to previous findings and demonstrate that initiation of clozapine leads to improved adherence over a 3-year period. Although widely recognised by clinicians as a barrier to clozapine use, previous poor adherence does not appear to justify avoiding clozapine treatment in patients who would otherwise be considered eligible.

